# Drug design for cyclin-dependent kinase 9 (CDK9) inhibitors *in silico*

**DOI:** 10.1016/j.bbrep.2025.101988

**Published:** 2025-03-28

**Authors:** Kaori Asamitsu, Takatsugu Hirokawa, Takashi Okamoto

**Affiliations:** a Department of Neurocognitive Science, Institute of Brain Science, Nagoya City University, Graduate School of Medical Sciences, Nagoya, Aichi, Japan; b Division of Biomedical Science, Faculty of Medicine, University of Tsukuba, Tsukuba, Ibaragi, Japan; c Transborder Medical Research Center, University of Tsukuba, Tsukuba, Ibaragi, Japan

**Keywords:** Cyclin dependent kinase (CDK) 9, Kinase inhibitor, In silico drug design, Molecular docking

## Abstract

Despite the potential of cyclin-dependent kinase 9 (CDK9) as a novel target for various malignancies and HIV replication in infected cells, no effective inhibitors have been developed. In the preceding study, we deciphered a hidden cavity in CDK9 upon molecular dynamics (MD) simulation of the CDK9/CyclinT1/Tat trimolecular complex. This cavity is located near the CDK9 ATP pocket (continuous cavity I, CCI) and extends to the cyclin T1 (CycT1) contact surface (CCII and CCIII). In this study, we searched for compounds similar to previously identified CDK9 inhibitors using cheminformatics to identify compounds that are better suited to this hidden cavity. We identified compounds that effectively targeted CCII and CCIII of CDK9. We confirmed their inhibitory effects on the CDK9/CycT1 complex *in vitro*. As these inhibitory compounds target only a portion (CCII and CCIII cavities) of CDK9, we examined their combinatorial effects with the known CDK inhibitor **BS-181**. As expected, this combination exerted an additive inhibitory effect on CDK9 expression. These findings confirm the presence of a CDK9 hidden cavity that was revealed transiently by MD simulations, thus providing promising evidence for the development of CDK9 inhibitors.

## Highlights

• CDK9 is a feasible target for HIV, but no effective inhibitors have been developed.
• We previously reported a hidden cavity linking the ATP- and cyclin T1-binding sites of CDK9.
• We found novel compounds targeting this hidden cavity.
• These compounds inhibited CDK9 and exhibited more than additive effects with BS-181.
• Discovering compounds for this cavity opens new potential for better CDK9 inhibitors.

## 1 Introduction

Cyclin-dependent kinase 9 (CDK9) is crucial for several genes involved in cancer progression [1,2] and HIV transcription in infected cells [3,4]. Interestingly, HIV-encoded Tat protein activates HIV transcription through direct interactions with cyclin T1 (CycT1), a crucial regulator of CDK9 [[5], [6], [7], [8], [9], [10]]. In the absence of Tat, viral transcription is incomplete and terminates after formation of the transactivation response (TAR) RNA sequence that is the target of the HIV-1 Tat protein [11,12]. Host positive transcription elongation factor b (P-TEFb) that contains CycT1 and CDK9 is recruited to initiate viral transcripts containing TAR in the presence of Tat through the Tat-CycT1 interaction [[5], [6], [7],^9^,^10^]. As Tat binds to the bulge and cycT1 binds to the loop of TAR RNA, a virus-specific transactivation complex containing Tat-TAR RNA-P-TEFb is formed [6,7]. This complex specifically phosphorylates the serine 2 residues of the RNA polymerase II (RNAP II) C-terminal domain, 5,6-dichlorobenzimidazole-1-β-d-ribofuranoside (DRB) sensitivity-inducing factor (DSIF), and negative elongation factor (NELF) [10]. NELF was shown to dissociate from RNAP II, thus converting DSIF protein into another conformation, so that transcriptional elongation by RNAP II is facilitated [10].

Various studies have indicated that CDK9 is a molecular determinant for the life cycle of HIV-1 and its latency [13,14]. For example, primary CD4 (+) T cells harboring latent HIV-1 contained the reduced level of CycT1 and CDK9 hypophosphorylated at Thr186 [15]. Thus, HIV replication and its gene expression is tightly regulated by P-TEFb, more specifically the CDK9 activity [16], and CDK9 inhibitors such as flavopiridol and FIT109 have been demonstrated to inhibit its expression [17,18]. These results highlight the essential role of the Tat-TAR RNA-P-TEFb axis in breaking and silencing HIV latency.

Recent studies have reported that CDK9 undergoes conformational changes upon Tat binding and that its activity is regulated [[19], [20], [21], [22]]. Particularly, crystal structure analyses and molecular dynamics (MD) simulations of the P-TEFb/Tat complex revealed that Tat-binding causes specific local conformational changes in CDK9, followed by the reorganization of the ATP pocket [[19], [20], [21], [22]]. The structure of CDK9 is characterized by a typical kinase structure in the N-terminal lobe (residues 16–108) consisting of five β-sheets and a characteristic α-helix containing PITALRE and the C-terminal lobe (residues 109–330) containing primarily an α-helix [23]. The CDK has characteristic AA sequences, such as PITALRE for CDK9, that are well conserved among different species. These sequences appear to direct distinct Cyclin proteins and thus specify activities of relevant CDKs. The α-helical region of the CDK9 N-terminal lobe is known to regulate the interaction between CDK9 and CycT1 [23]. Then, the ATP-binding motif or pocket, that is highly conserved among various CDKs, creates a cleft between the N- and C-terminal lobes [23]. Many CDK9 inhibitors have been designed to competitively bind to this ATP-binding pocket and inhibit CDK9 activity by blocking ATP binding [1,24]. However, as the ATP-binding motif is conserved throughout the CDK family, these inhibitors are not specific to CDK9. To overcome this shortcoming, alternative approaches have been devised to exploit subtle structural differences in the ATP-binding pocket of CDK9. One of these is DRB that blocks the ATP-binding site of CDK9 by halogen binding, thereby causing a conformational change in the glycine-rich loop of CDK9 [25]. Additionally, flavopiridol is known to bind the CDK9 ATP pocket and induce structural changes that conforms the binding site for this inhibitor [23].

Recent drug development has been successful by utilizing the result of MD simulation of 3D structures obtained by X-ray crystallography. It was then found that subtle molecular conformational changes should allow better compound fitting for pharmacophores of target molecules. For example, Filomia et al. [26] used MD simulations of p38 MAP kinase to decipher hidden molecular cavities suitable for effective inhibitors. Similarly, during the development of CDK9 inhibitors, our MD simulations of Tat/P-TEFb compared to those of P-TEFb revealed a specific Tat-binding-dependent cavity in CDK9 that could be a potential target for creating new inhibitors [22].

In this study, we utilized cheminformatics approaches to identify compounds structurally analogous to previously characterized CDK9 inhibitors. The primary objective was to explore the functional significance of the Tat-induced cavity within CDK9 by identifying molecules with enhanced compatibility for this concealed binding pocket, thereby informing the design of novel CDK9 inhibitors.

## 2 Materials and methods

### 2.1 In silico structural similarity search of 1805

We conducted a structural search using the JChem Base provided by Chemaxon (https://docs.chemaxon.com/display/docs/jchem-base_index.md) to collect available compounds that are similar to **1805** [22], employing both fingerprint similarity and substructure search tools. For the compound library, we used an integrated database containing compound data from various suppliers (https://www.namiki-s.co.jp/compound/database.php). Chemically hashed fingerprints were utilized for the similarity search. These fingerprints were created by identifying all linear patterns and rings within the chemical structure, and the fingerprint bits were assigned via a hashing function. The similarity between molecules was assessed using the Tanimoto coefficient, which measures the extent of similarity between two compounds. A Tanimoto coefficient (Tc) cut-off value of 0.6 was applied. The final selection was based on the following criteria: (1) Compounds identified through the similarity and substructure search were filtered by molecular weight (MW) of 300 Da or less, (2) Substituents from the amide group of 2-(4-aminophenyl) acetic acid, a key scaffold, were prioritized, considering structure-activity relationships from our previous studies [22], and (3) Compounds that are available in terms of stock status, quantity, and other logistical factors were selected.

### 2.2 Docking and MD simulations of hit compounds

In our previous study, we discovered that the CDK9 hidden cavity is formed through MD simulations of the X-ray complex structure of CDK9/CycT1 in the presence of Tat binding [20]. We defined this structural state as the “CDK9 model” and have since utilized it for the exploration of ligands targeting the hidden cavity. Through docking models and activity assays, we have demonstrated that **127** possesses a binding affinity for the hidden cavity [22].

The CDK9 model bound to **127** [22] was refined for docking simulations using the Protein Preparation Wizard Script in Maestro (Schrödinger, LLC, New York, NY, USA). Ionization and energy minimization of hit compounds were performed using the OPLS3e force field in the LigPrep Script in Maestro. The 20 x 20 × 20 Å grid box for the hidden cavity was defined by the center of mass of the binding position of **127** (S1 Fig). Docking simulations of hit compounds to the hidden cavity within CDK9 were performed using the Glide XP docking program with the common substructure constraint of **127** (root mean square deviation (RMSD) tolerance was set at 2 Å) [27,28] (Schrödinger, LLC, New York, NY, USA). As a significance criterion for the docking score, compounds with a score of −5 kcal/mol or lower, corresponding to a Kd value of 10 μM, were identified as hits and evaluated. As a post-docking process, the best docking score pose for each hit compound was evaluated using MD simulation to assess the stability of its binding. The MD simulation of the complex structure of CDK9 and the hit compound was conducted using gDesmond36 version 5.7 (Schrödinger, LLC, New York, NY, USA) with the OPLS3e force field. The initial structures were refined through the Protein Preparation Wizard in Maestro and solvated in SPC water with 0.15 M NaCl. After minimizing and relaxing the model, the production MD phase involved three independent 200 ns simulations, each with different initial velocities, in an isothermal-isobaric (NPT) ensemble at 300 K and 1 bar, employing a Nose–Hoover thermostat. Long-range electrostatic interactions were calculated using the Smooth Particle Mesh Ewald method. All system setup steps were completed in Maestro. Trajectory coordinates were recorded at 10 ps intervals. The resulting trajectory was analyzed using the Simulation Interaction Diagram (Schrödinger, LLC, New York, NY, USA) to calculate the RMSD of the compound from an initial docking pose. The binding free energy of the hit compound was calculated using MM-GBSA (Schrödinger, LLC) every 1 ns out of 200 ns of each production run.

### 2.3 Docking simulation of BS-181 within the hidden cavity of CDK9

The CDK9 model without **127** [22] was refined for docking simulations using the Protein Preparation Wizard Script in Maestro. Ionization and energy minimization of **BS-181** were performed using the OPLS3e force field in the LigPrep Script in Maestro. The 20 x 20 × 20 Å grid box for **BS-181** was defined by the center of mass of the ATP-binding pocket. To define the ATP-binding pocket, the CDK9 model was superimposed onto the crystal structure of human CDK9/cycT1 in a complex with ATP (PDB: 3BLQ). Docking simulations of **BS-181** to ATP-binding pocket within CDK9 were performed using the Glide Induced Fit Docking program (Schrödinger, LLC, New York, NY, USA) with default parameters. The lowest docking score pose was selected.

### 2.4 Kinase inhibition assays

*In vitro* kinase assays were performed as previously described [22]. Briefly, recombinant proteins were obtained using the baculovirus expression system and purified using glutathione sepharose chromatography. For CDK7/Cyclin H/MAT1, full-length human CDK7 (amino acids 1–346) was co-expressed with full-length Cyclin H (amino acids 1–323) and full-length MAT1 (amino acids 1–309) as an N-terminal GST-fusion protein. GST-CDK7 was subsequently purified using glutathione sepharose chromatography. For CDK9/CycT1, full-length human CDK9 (amino acids 1–372) was co-expressed as an N-terminal GST-fusion protein with full-length His-CycT1 (amino acids 1–726), and GST-CDK9 was subsequently purified using glutathione sepharose chromatography. These recombinant proteins were obtained from Carna biosciences (Kobe, Japan). (S2 and S3 Figs).

The test compounds were dissolved in dimethyl sulfoxide and diluted to a concentration of 100-fold higher than the target concentration, unless otherwise stated. This solution was further diluted 25-fold with assay buffer (20 mM HEPES [pH 7.5], 0.01 % Triton X-100, and 1 mM DTT) to create a 4 × compound solution. Reference compounds for the assay controls were prepared in a similar manner.

### 2.5 Off-chip mobility shift assay and data analysis

Kinase inhibition profiles were determined using an off-chip mobility shift assay (MSA) provided by Carna Biosciences (Kobe, Japan) [29]. This assay uses recombinant human CDK/cyclin complexes and their corresponding substrates at various ATP concentrations. S1 Table provides details of the protein molecules, substrates, and ATP concentrations used during the assay. Phosphorylation of the peptide substrate was measured in the presence or absence of test compound(s). Details of the assay procedure have been reported previously [22,29]. In summary, a 4 × substrate/ATP/metal solution was prepared using a kit buffer (20 mM HEPES, 0.01 % Triton X-100, 5 mM DTT, pH 7.5), and 2 × kinase solution containing the CDK/Cyc complex was prepared using the assay buffer. Five microliters of the 4 × compound solution, 5 μL of the 4 × substrate/ATP/metal solution, and 10 μL of the 2 × kinase solution were combined and incubated in a polypropylene 384-well microplate for either 1.5 or 5 h at room temperature. The reactions were terminated by adding 70 μL of termination Buffer (127 mM HEPES, 0.01 % Triton X-100, 26.7 mM EDTA-2Na, 1 % DMSO, pH 7.5). The reaction mixtures were then analyzed using the LabChip™ system (PerkinElmer), where the product and substrate peptide peaks were separated and quantified. Kinase activity was assessed based on the product ratio, calculated using formula (P/[P + S]), where P and S represent the peak heights of the product and substrate peptides, respectively. The reaction control (complete reaction mixture) was set to 0 % inhibition and background control (without enzyme) was set to 100 % inhibition. The percent inhibition of each test solution was calculated. All experiments were performed in duplicate and a positive control was used for assay verification. Relative kinase activity was reported as percent inhibition in accordance with off-chip MSA.

## 3 Results

### 3.1 In silico screening of compounds with CDK9 inhibitory activities

**Fig. 1:**
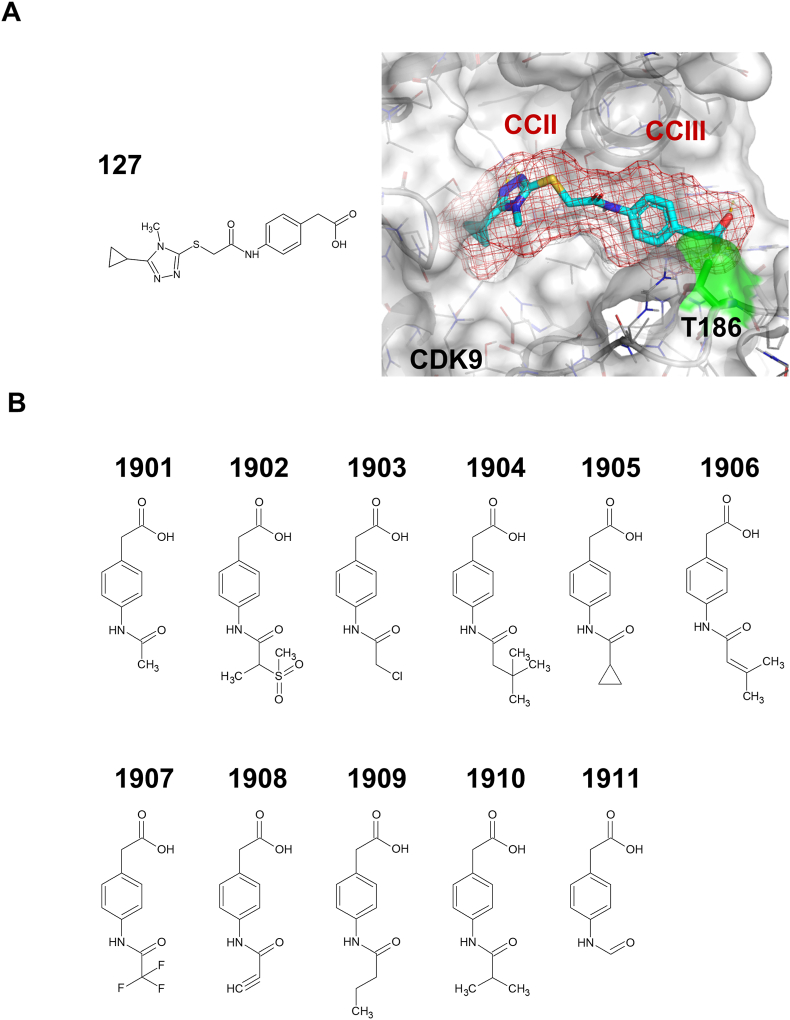
The CDK9 hidden cavity and 1805 derivatives. (A) The structure of **127** in complex with the CDK9 hidden cavity. The presence of CCII and CCIII [22] is shown with a red mesh surface. The position of CDK9 Thr-186 is highlighted in green-yellow. CDK9 Thr-186 and **127** are located within a 4 Å proximity. (B) Substructure derivatives of **1805**.

As previously reported, we identified the novel CDK9 inhibitor **127** and its subgroups **1804** and **1805** through *in silico* screening using the CDK9 hidden cavity as a pharmacophore [22]. The cavity is comprised of three interconnected cavities, including CCI that houses the ATP-binding site, CCIII that contains the Tat-binding site near the T-loop, and CCII that links the two functional regions. **127** fitted well into the CCII to CCIII portion of the hidden cavity (Fig. 1A). Several amino acids of CDK9 were identified to interact with **127**, among which Gln27, Thr62, and Thr186 are particularly important for its inhibitory action on CDK9 [22]. While Thr186 is known to play a pivotal role in regulating CDK9 activity, Gln27 and Thr62 may be involved in the specificity determination of CDKs. The substructural analogs **1804** and **1805** exclusively filled CCIII, indicating that CCIII may be the critical cavity for inhibiting CDK9 [22]. However, due to the small size of these molecules compared to that of **127**, their CDK9 inhibitory activity was relatively low.

To further expand the target substructures of CDK9, we have performed a structural search using the JChem Base (Chemaxon Ltd.) to collect available compounds similar to **1805**, employing both fingerprint similarity and substructure search tools. As a result, the similarity search (Tc ≥ 0.6 and MW ≤ 300 Da) and substructure search (MW ≤ 300 Da) yielded 56 (S4 Fig) and 132 (S5 Fig) compound candidates, respectively. We procured 11 commercially available compounds (Fig. 1B) and, considering the structure-activity relationships from our previous studies [22], subsequently evaluated their inhibitory effects of **1805** analogs with extended structures on CCII.

*In vitro* kinase assays were performed using the recombinant CDK9/CycT1 complex and CDK9 substrate with 0.01 mM ATP, and this is similar to the Km value of CDK9 (https://www.carnabio.com/english/product/search.cgi) at 100 μM concentration of compounds. **1903** and **1908** exhibited the highest inhibitory activities, as presented in Table 1 and S1 File. We then investigated the CDK9 inhibitory activity of these compounds by increasing the ATP concentration from 0.01 to 1.0 mM for examination of possible involvement of the CDK9 ATP pocket in its inhibition. The inhibitory effects of **1903** and **1908** on CDK9, compared to other compounds, were demonstrated even when ATP was present in excess (Table 1). These results suggested that the ATP-independent inhibition of **1903** and **1908** likely occurred by binding to CCIII rather than competing with CCI.

**Table 1:** Inhibitory effects of 1805 derivatives on CDK9.

	ATP 0.01 mM	ATP 1 mM
1901	0.6	0
1902	6.0	0.8
1903	45.6	25.3
1904	0.1	0
1905	4.5	0.3
1906	2.2	0
1907	12.3	8.2
1908	31.7	19.3
1909	0.7	0
1910	0	0
1911	0	0

∗ Apparently negative values for activity indicated no inhibitory activity.

We further tested the binding potential of **1903** to the CCIII pocket by docking and MD simulations. Docking results showed that **1903** could bind to CCIII similar to the binding mode of **127** to CCIII (S6 Fig). The docking score was −5.639 kcal/mol. This binding mode was evaluated three times for stability in solution by MD simulations. The results clearly show that one replica MD keeps the docking pose, and in the other two, the carbonyl group retains binding to the arginine residues around the CCIII pocket, albeit with fluctuations. The binding free energies based on simulations of Replica 1, 2, and 3 were −32.6532 (SD = 4.31), −33.0680 (SD = 5.28), and −32.2755 (SD = 4.76), respectively.

### 3.2 Effects of adding a known ATP-binding inhibitor (BS-181)

**Fig. 2:**
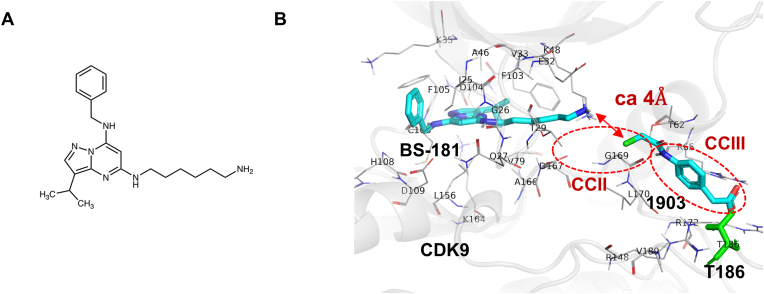
Predicted binding mode of 1903 and BS-181 (A) Chemical structure of **BS-181**. (B) Predicted binding mode of **1903** and **BS-181** by docking simulation. The model structure in the figure shows the CDK9-**1903** complex superimposed on the CDK9-**BS-181** complex model, using the CDK9 structure as a reference. Docking simulations were conducted independently for 1903 and BS-181. Docking score of **1903** and **BS-181** are −5.15 and −8.76 kcal/mol, respectively.

To further investigate the mode of CDK9 inhibition by **1903**/**1908**, we examined its combined effects with a known kinase inhibitor, **BS-181** (Fig. 2A), that was obtained by molecular docking based on the 3D structure of CDK7 [30]. **BS-181** competitively binds to the ATP-binding site of CDK7, thereby inhibiting its ATP binding and kinase activity. The effect of **BS-181** on CDK9 activity was examined *in vitro* (Table 2 and S2 File). BS-181 also inhibited CDK9; however, this effect was observed at higher concentrations than that for CDK7. These results suggest that the effect of **BS-181** on CDK9 may be due to competition for ATP binding.

**Table 2:** The extents of CDK9 and CDK7 inhibition by BS-181.

	0.01 μM	0.1 μM	1 μM	10 μM	100 μM
CDK9/CycT1	1.0	10.5	55.5	93.7	99.4
CDK7/CycH/MAT1	50.2	94.1	104.1	100.6	101.8

We examined the effects of **1903** and **1908** on CDK9 kinase activity in the presence of low doses of **BS-181** (Table 3 and S3 File). The inhibitory activities of both compounds were largely independent of the effect of **BS-181**, although significant synergism was observed, particularly at low concentrations. For example, although **1903** alone at a concentration of 1.0 μM exhibited 1.1 % inhibition, addition of **BS-181** at 0.1 μM augmented the inhibition to 14.7 %. Additionally, while 11 % CDK9 inhibition was observed with 0.1 μM **BS-181**, 38 % and 77 % CDK9 inhibition was observed when **1903** was added at 10 and 100 μM, respectively.

**Table 3:** Combinatorial effects on CDK9/CycT1 inhibition.

	BS181		
	0 μM	0.01 μM	0.1 μM
1903, 0 μM	–	0	11
1 μM	1.1	1.4	14.7
10 μM	16.6	17.9	38
100 μM	59.2	62.1	76.9

1908, 0 μM	–	0	11
1 μM	5.1	6.7	17.8
10 μM	16.2	17.8	28.7
100 μM	28.2	30.7	43.1

∗ Apparently negative values for activity indicated no inhibitory activity.

Finally, as presented in Fig. 2B, molecular docking simulations revealed the complex configuration of CDK9 with **BS-181** and **1903**. **BS-181** was positioned near the ATP-binding pocket of CDK9, while **1903** was located close to CCIII. The distance between **1903** and CDK9 Thr186 was measured at approximately 4 Å. Similarly, the distance between **127** and Thr186 was approximately 4 Å (Fig. 1B). These observations suggest that both **1903** and **127** may regulate CDK9 activity by interacting with Thr186. Two small compounds were aligned within the hidden CDK9 cavity without any interference. As there is a small gap of approximately 4 Å, chemical ligation of these two compounds should provide further efficient CDK9 inhibition.

## 4 Discussion

Transcriptional elongation is a promising treatment target in cancer, leukemia, and HIV infection [[1], [2], [3], [4]]. This process is primarily regulated by P-TEFb, a protein complex containing CDK9 and CycT1. First-generation inhibitors including flavopiridol and seliciclib, often known as “pan-CDK” inhibitors, target the ATP-binding sites of CDKs and exhibit suboptimal therapeutic effects [31,32]. As the ATP-binding region is conserved across the entire CDK family, these inhibitors lack specificity for CDK9. These findings should provide useful information for developing novel CDK9 inhibitors.

In this study, using *in silico* analysis, we found **1903** and **1908** that specifically bind to the CCII and CCIII regions of the hidden cavity in CDK9. We confirmed the CDK9 inhibitory activity using *in vitro* kinase assays. When combined with the known ATP-binding inhibitor **BS-181**, these compounds exhibited additive effects on CDK9 inhibition. Molecular docking simulations revealed that **BS-181** and **1903** were positioned without steric hindrance. These findings suggested that **BS-181** and **1903** or **1908** act synergistically to inhibit CDK9 expression. The finding that neither **1903** nor **1908** antagonized CDK9 inhibition in the presence of **BS-181** further supports the significance of CCII and CCIII, reconfirming the reality of the CDK9 hidden cavity and its applicability as a target structure for screening CDK9 inhibitors.

Small-molecule compounds that inhibit protein kinases are considered potential therapeutics for several diseases [33]. Dar and Shokat [34] classified these inhibitors into three categories that included type I inhibitors that bind to the active ATP pocket, type II inhibitors that bind to an inactive conformation (DFG-out), and type III inhibitors that act allosterically. In addition to type I, type II, and type III inhibitors that bind away from ATP sites, Gavrin and Saiah [35] further divided type III allosteric inhibitors into two subgroups that included revised type III binding near the ATP-binding pocket and type IV binding outside of the lobes. Studies investigating kinase inhibitors have redesigned type III inhibitors to reduce promiscuous toxicity and avoid drug resistance caused by mutation of the ATP-binding site [36]. The CDK9 inhibitors that utilize the CDK9 hidden cavity described in this report are hybrids of types I and III. Recently, an *in silico* screening study utilizing flavopiridol as a template identified novel CDK9 inhibitors, which target the ATP-binding site and are considered to function as a Type I inhibitor [37]. Their study is restricted to the ATP-binding site, corresponding to CC1, which differs from CCII and CCIII of CDK9 hidden cavity. The compounds we have found here are expected to act as kinase inhibitors via novel mechanisms of action.

In addition to HIV treatment, CDK9 inhibitors are promising therapeutics against cancers and leukemia, with specific features that target transcriptional elongation in malignant cells and viral transcription in HIV-infected cells [[1], [2], [3], [4],^38^]. However, the lack of selectivity and adverse effects of current inhibitors, such as flavopiridol and dinaciclib, have hindered their applicability in clinical settings [1]. These findings provide promising evidence for the development of novel CDK9 inhibitors. Future efforts should focus on the continued search for inhibitors that bind to the hidden cavity of CDK9, including the synthesis of compounds that link CCI to CCII and CCIII.

## CRediT authorship contribution statement

Kaori Asamitsu: Conceptualization, Investigation, Writing - Original Draft, Writing - Review & Editing, Funding acquisition Takatsugu Hirokawa: Conceptualization, Investigation, Writing - Original Draft, Writing - Review & Editing, Visualization, Funding acquisition Takashi Okamoto: Conceptualization, Writing - Original Draft, Writing - Review & Editing.

## Ethics approval

Not applicable.

## Consent for publication

Not applicable.

## Data availability

The datasets analyzed during the current study are available from the corresponding author on request.

## Funding

This work was supported by JSPS KAKENHI Grant Number JP17K08638 to KA and the Research Support Project for Life Science and Drug Discovery [Basis for Supporting Innovative Drug Discovery and Life Science Research (BINDS)] (grant no. JP24ama121029j0003) of the Japan Agency for Medical Research and Development (AMED) to TH.

## Declaration of competing interest

The authors declare that they have no competing interests to report.
