# Supplementary material for: Drug design for cyclin-dependent kinase 9 (CDK9) inhibitors in silico
Source: Biochem Biophys Rep. 2025 Mar 28;42:101988. doi: 10.1016/j.bbrep.2025.101988 (PMC11995094; doi:10.1016/j.bbrep.2025.101988)
Supplement: S1_fig [file mmc1.pdf]

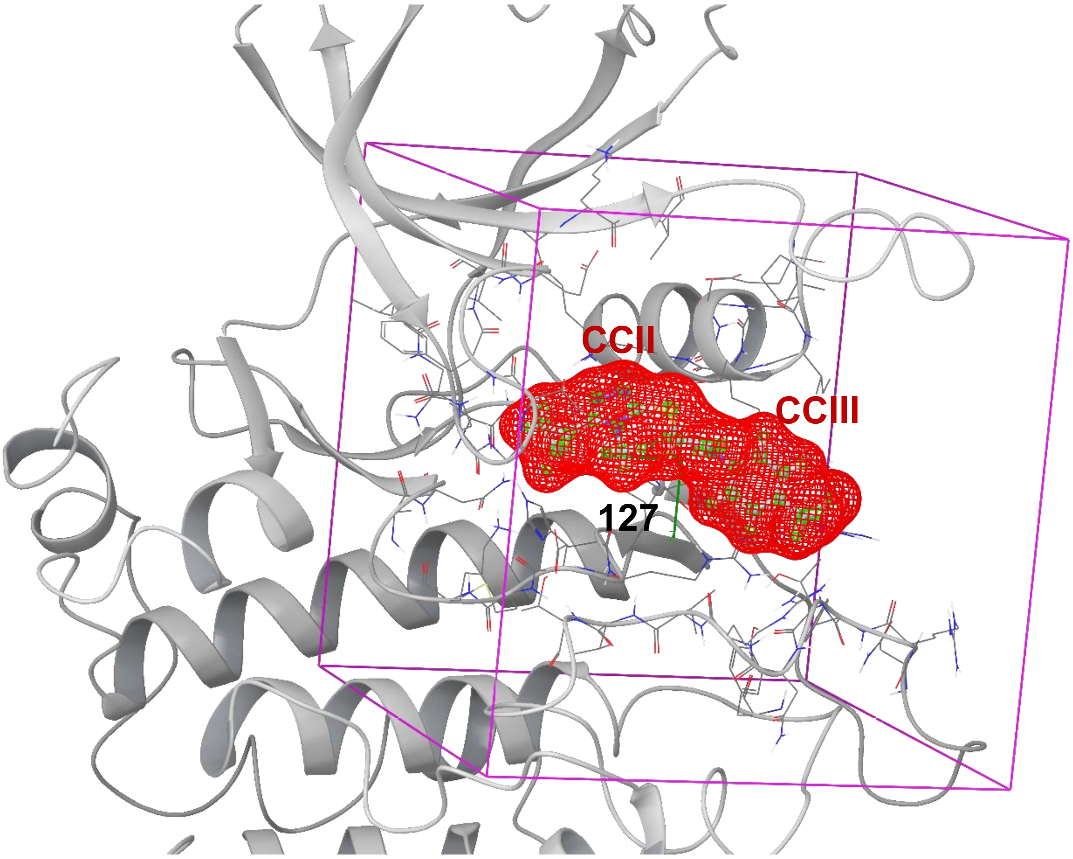

Figure S1. Definition of the grid box for the binding site position of compound 1906. The grid boxe for compound **1903** was defined by the center of mass the binding position of compound **127**. Default parameters were used, specifying a grid box size of 20 x 20 x 20 Å.
