# Supplementary material for: Drug design for cyclin-dependent kinase 9 (CDK9) inhibitors in silico
Source: Biochem Biophys Rep. 2025 Mar 28;42:101988. doi: 10.1016/j.bbrep.2025.101988 (PMC11995094; doi:10.1016/j.bbrep.2025.101988)
Supplement: S1_Table [file mmc2.pdf]

S1 Table. Reaction conditions of the *in vitro* kinase assay.

Kinase		Substrate		ATP	
Kinase	Conc.(ng/ml)	Substrate	Conc. (nM)	Conc. (mM) for Km value	Conc. (mM)
CDK7/CycH/MAT1	800	CTD3 peptide	1000	0.05	
CDK9/CycT1	1000	CDK9 substrate	1000	0.01	
CDK9/CycT1	1000	CDK9 substrate	1000		1

*Mg2+ at 5mM concentration was used in all the incubation and reaction was performed 5hrs. Staurosporine was used as a positive control in this assay.
