# Supplementary material for: Drug design for cyclin-dependent kinase 9 (CDK9) inhibitors in silico
Source: Biochem Biophys Rep. 2025 Mar 28;42:101988. doi: 10.1016/j.bbrep.2025.101988 (PMC11995094; doi:10.1016/j.bbrep.2025.101988)
Supplement: S2_Fig [file mmc3.pdf]

Carna Biosciences, Inc.
3F, BMA, 1-5-5, Minatojima-Minamimachi,
Chuo-ku, Kobe 650-0047 Japan
PHONE : +81-78-302-7091 FAX : +81-78-302-7086
e-mail : info@carnabio.com
URL : http://www.carnabio.com

Product Information

CDK9／CycT1

Product Number : **04-110**
Lot : **12CBS-0616 A**

Product description

Full-length human CDK9 [1-372(end) amino acids of accession number NP_001252.1] was co-expressed as N-terminal GST-fusion protein (70 kDa) with His-CyclinT1 [1-726(end) amino acids of accession number NP_001231.2] using baculovirus expression system. GST-CDK9 was purified by using glutathione sepharose chromatography.

Contents :

682 μg total protein / Vial

Concentration :

626 μg/mL in storage buffer

Concentration was determined by Bradford method with BSA standard.

Storage buffer:

50 mM Tris-HCl, 150 mM NaCl, 0.05% Brij35, 1 mM DTT, 10% glycerol, pH7.5

Storage and Handling:

Store at -80℃. Avoid repeating freeze-thaws.
Recommended validity date: One year from the date of shipment.
The expiration date may be extended only if test results are acceptable for your intended use.

SDS-PAGE

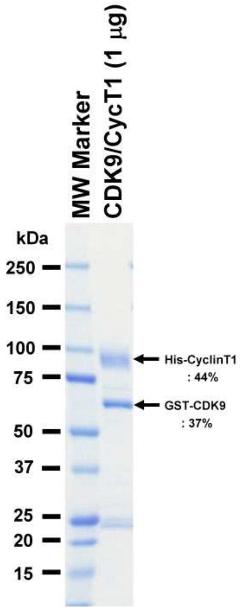

Purity: 81 %

The purity was assessed by SDS-PAGE/CBB staining.

Activity data

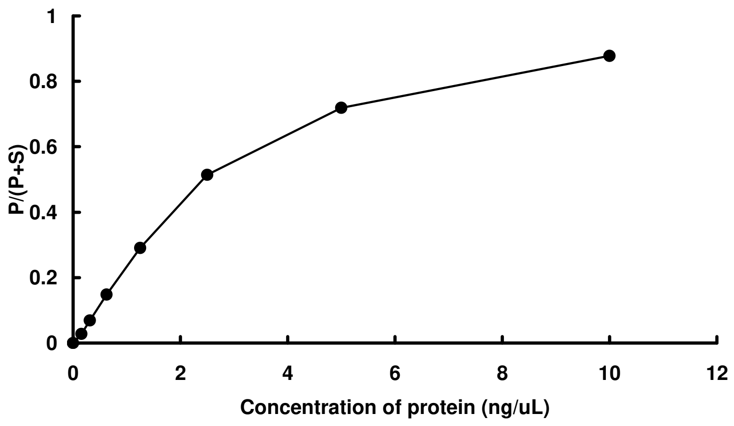

The activity was measured by off-chip mobility shift assay(MSA). The enzyme was incubated with fluorecence-labeled substrate and Mg(or Mn)/ATP. The phosphorylated and unphosphorylated substrates were separated and detected by MSA device.

Substrate : CDK9 substrate

ATP : 100 μM

Note: This enzyme is 'Research Purpose Use Only.'

Warranty information concerning our products can be found on our website at http://www.carnabio.com/english/disclaimer.html.

Figure S2. Product information of CDK9/CycT1
