# Supplementary material for: Drug design for cyclin-dependent kinase 9 (CDK9) inhibitors in silico
Source: Biochem Biophys Rep. 2025 Mar 28;42:101988. doi: 10.1016/j.bbrep.2025.101988 (PMC11995094; doi:10.1016/j.bbrep.2025.101988)
Supplement: S4_fig [file mmc7.pdf]

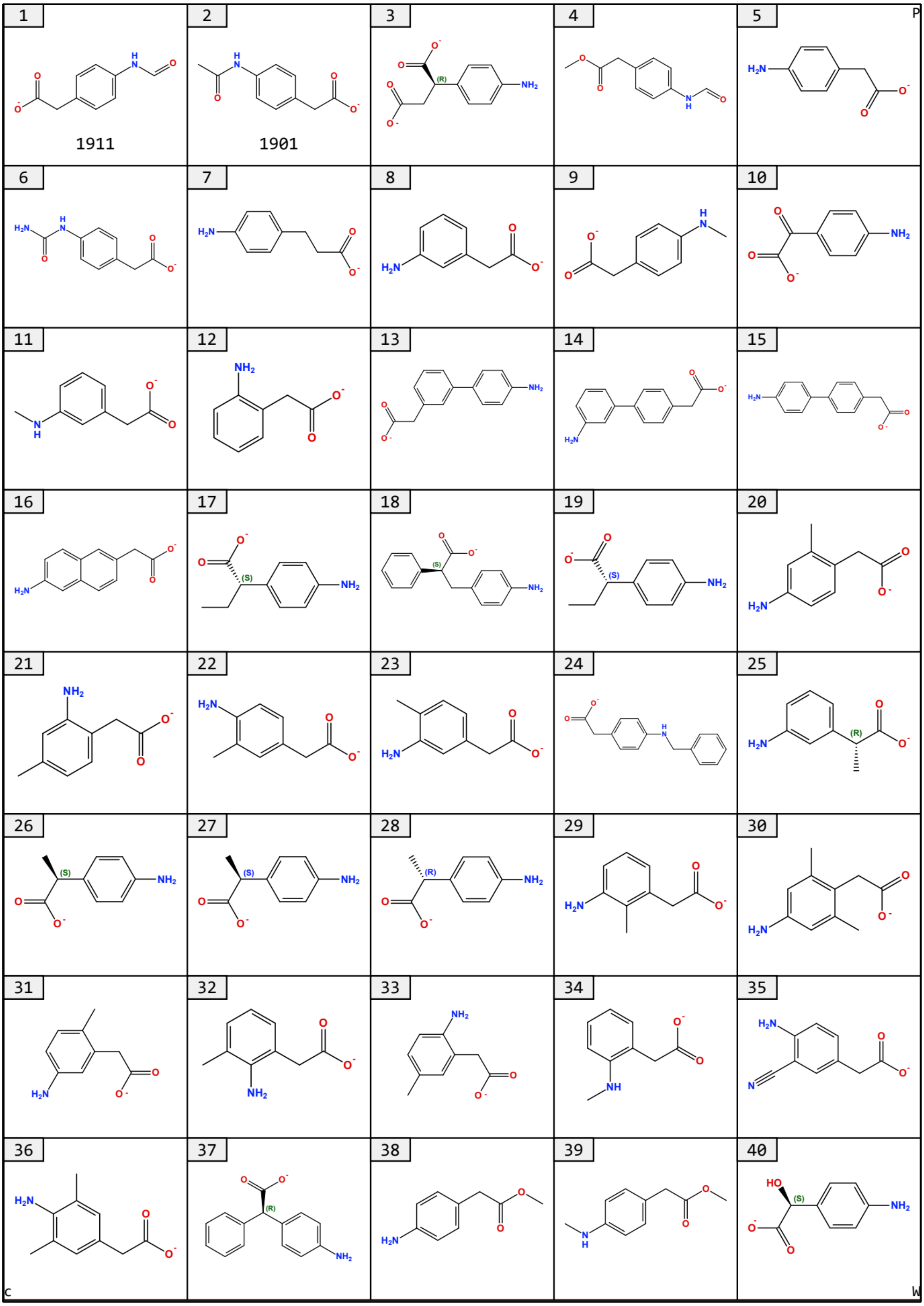

Figure S4. Similarity search results against compound 1805. Among the hit compounds, 56 compounds were identified that had a molecular weight of less than 300 Daltons and a Tanimoto coefficient higher than 0.6.

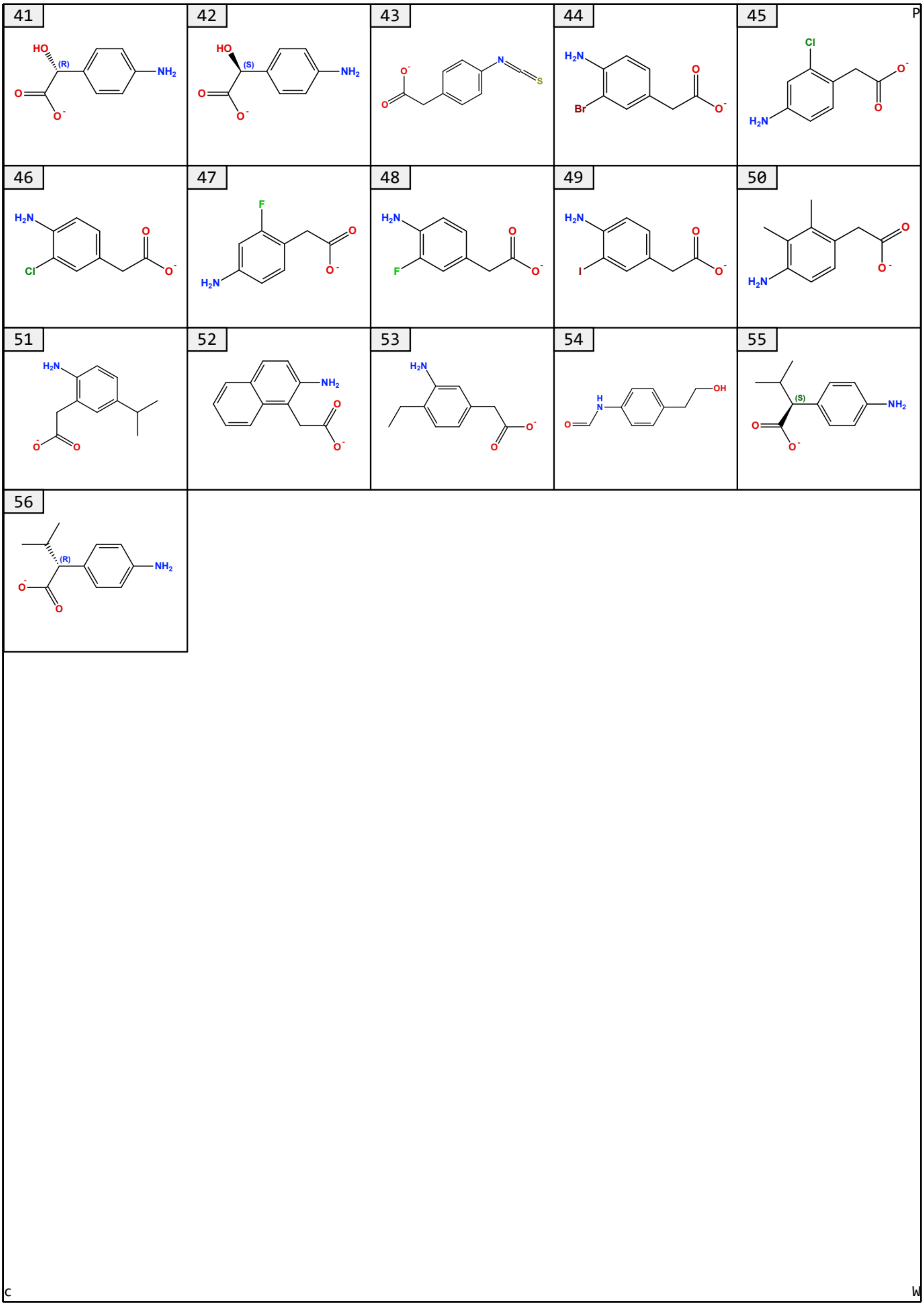

Figure S4. Continued.
