# Supplementary material for: Drug design for cyclin-dependent kinase 9 (CDK9) inhibitors in silico
Source: Biochem Biophys Rep. 2025 Mar 28;42:101988. doi: 10.1016/j.bbrep.2025.101988 (PMC11995094; doi:10.1016/j.bbrep.2025.101988)
Supplement: S5_fig [file mmc9.pdf]

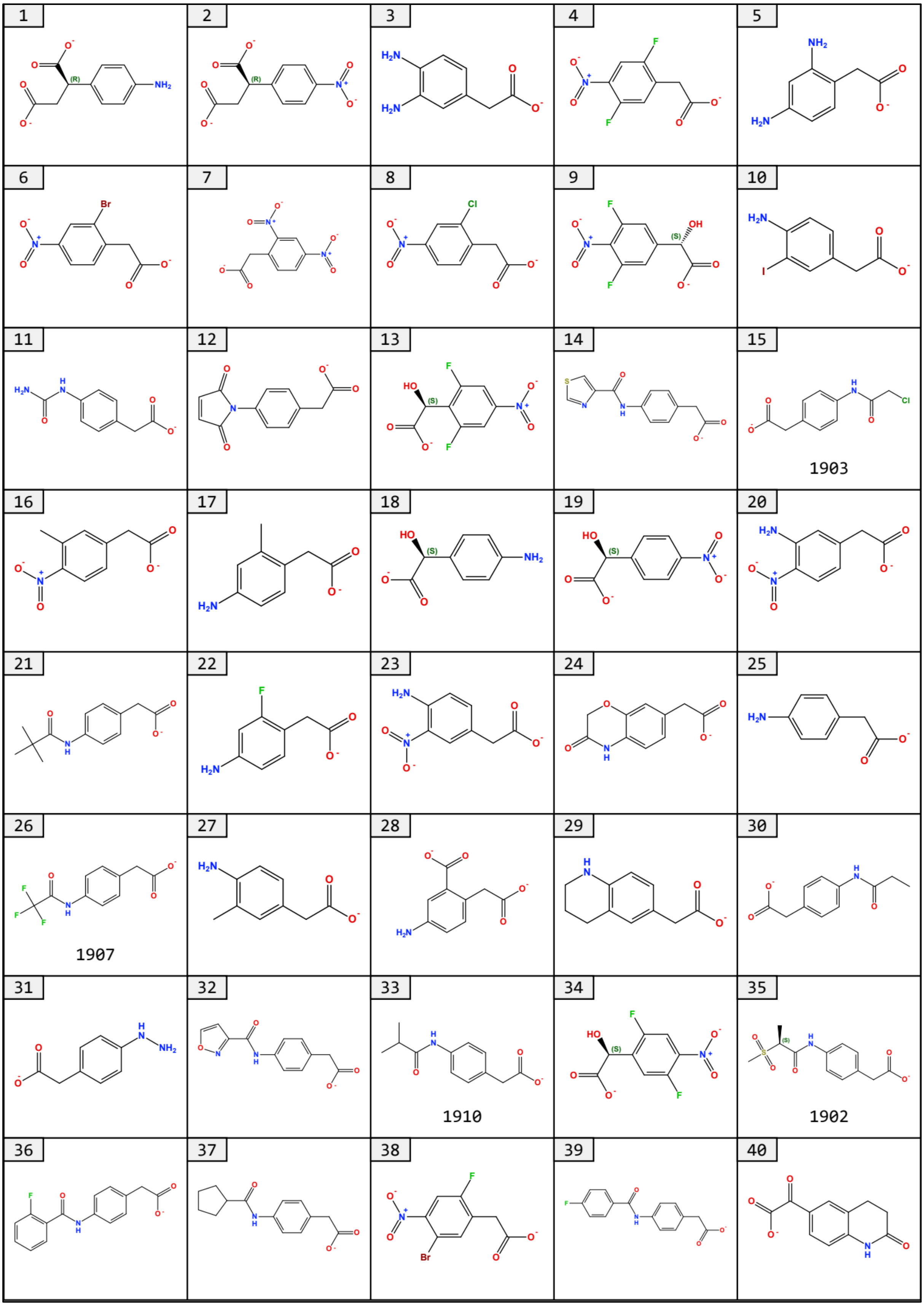

Figure S5. Substructure search results against compound 1805. Among the hit compounds, 132 compounds were identified with molecular weights less than 300 Daltons.

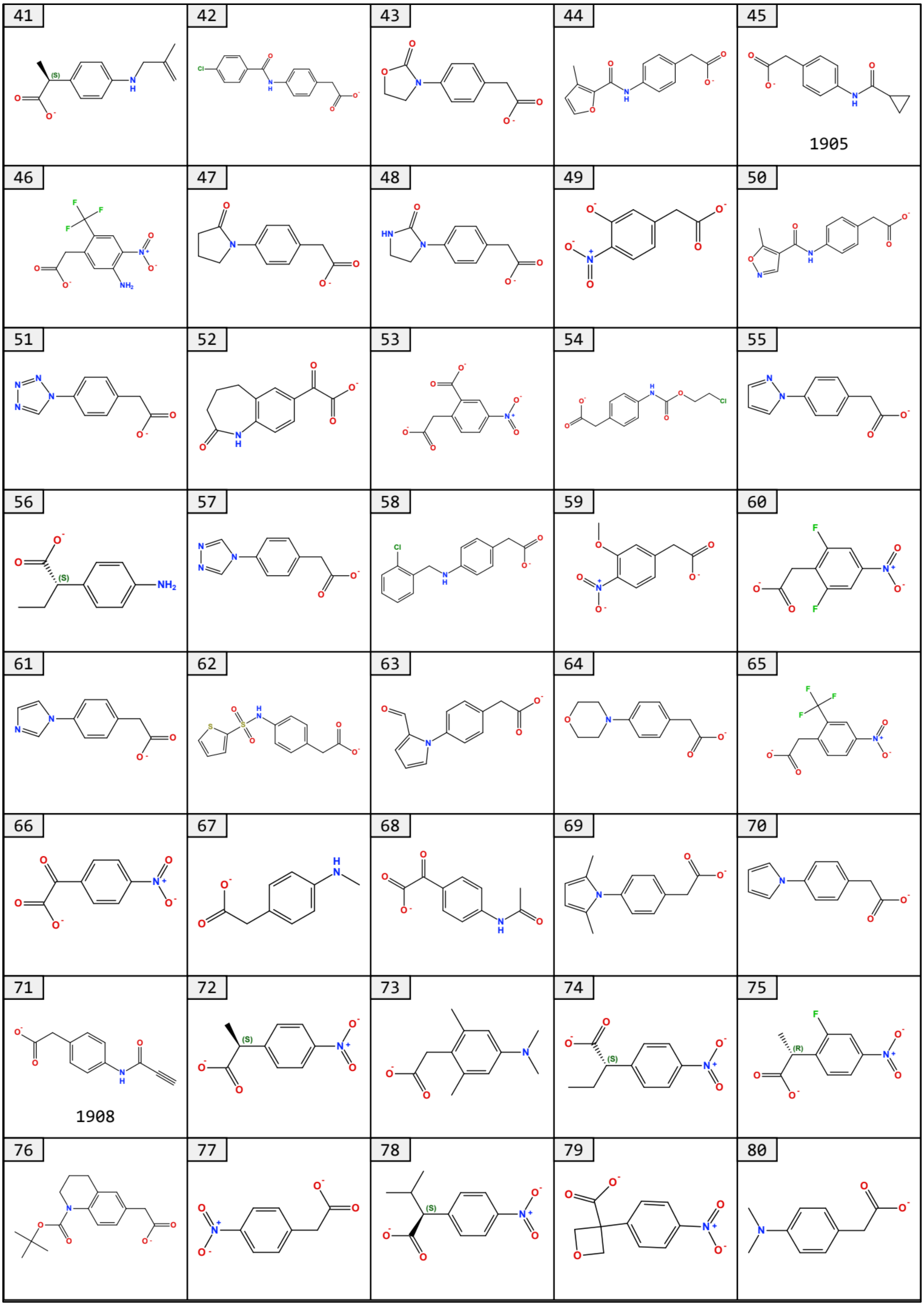

Figure S5. Continued.

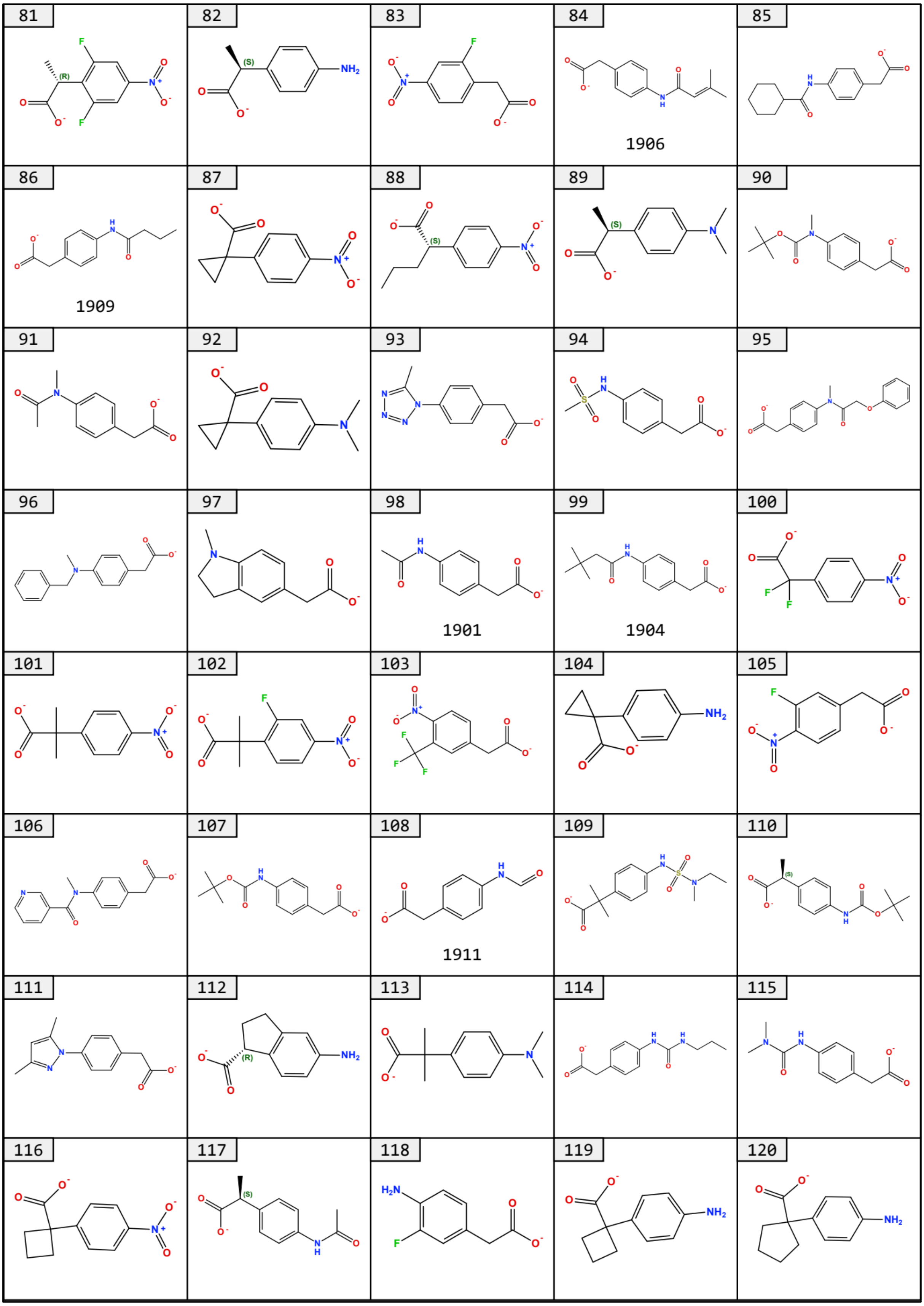

Figure S3. Continued.

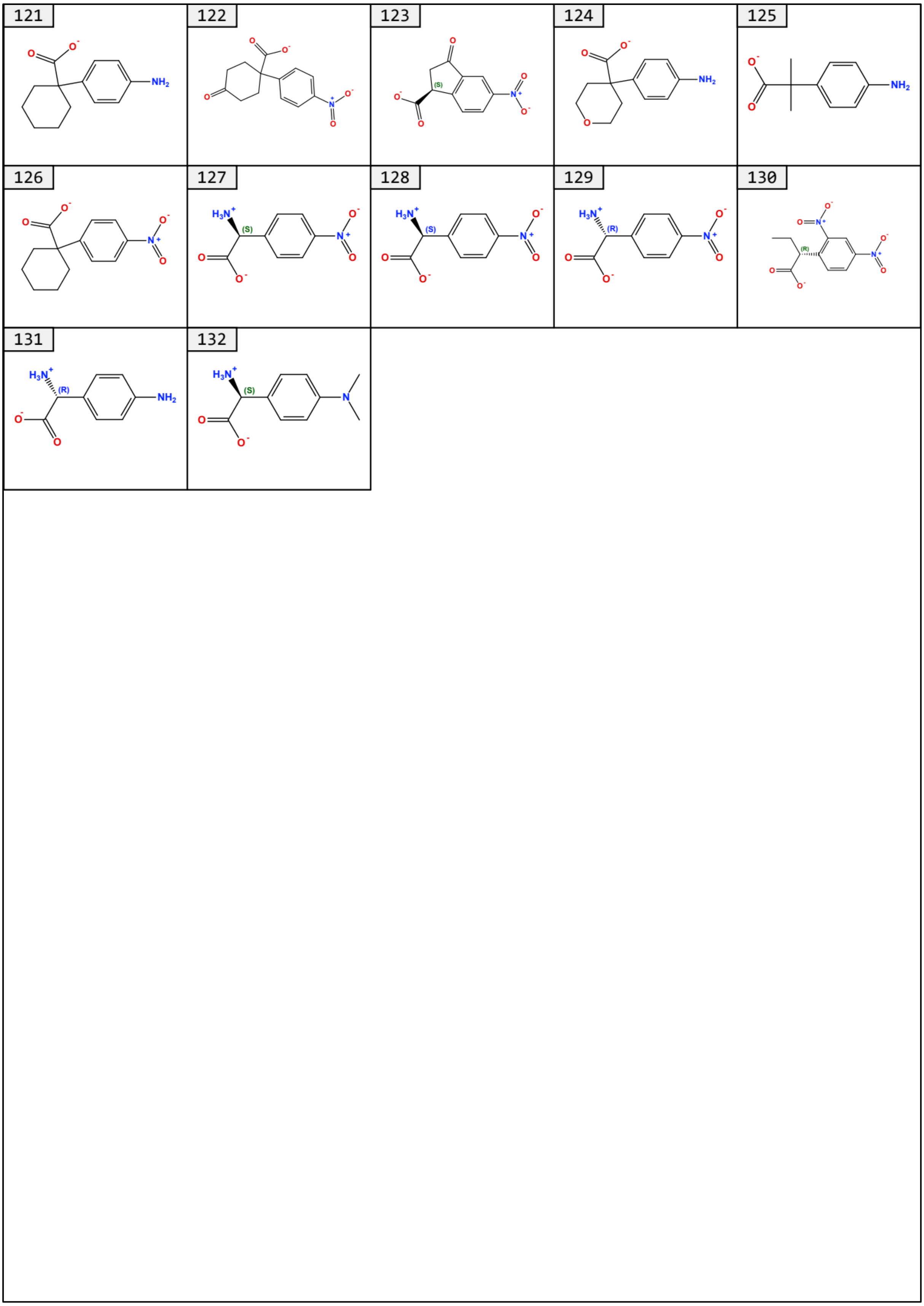

Figure S3. Continued.
