# Supplementary material for: Drug design for cyclin-dependent kinase 9 (CDK9) inhibitors in silico
Source: Biochem Biophys Rep. 2025 Mar 28;42:101988. doi: 10.1016/j.bbrep.2025.101988 (PMC11995094; doi:10.1016/j.bbrep.2025.101988)
Supplement: S6_fig [file mmc10.pdf]

A

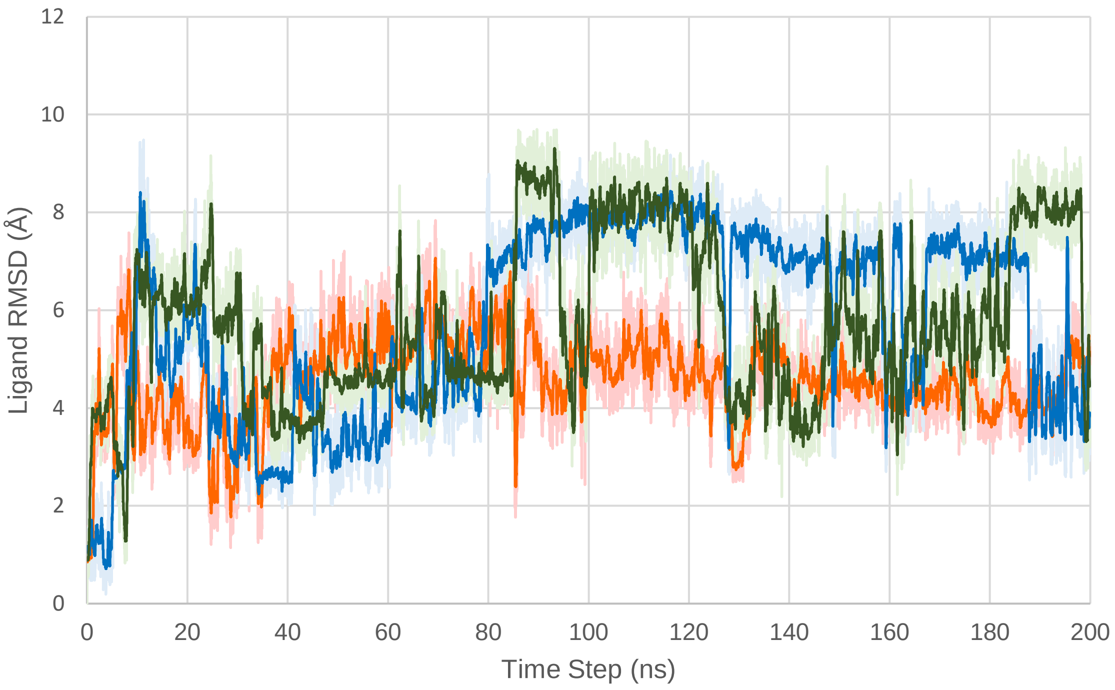

B

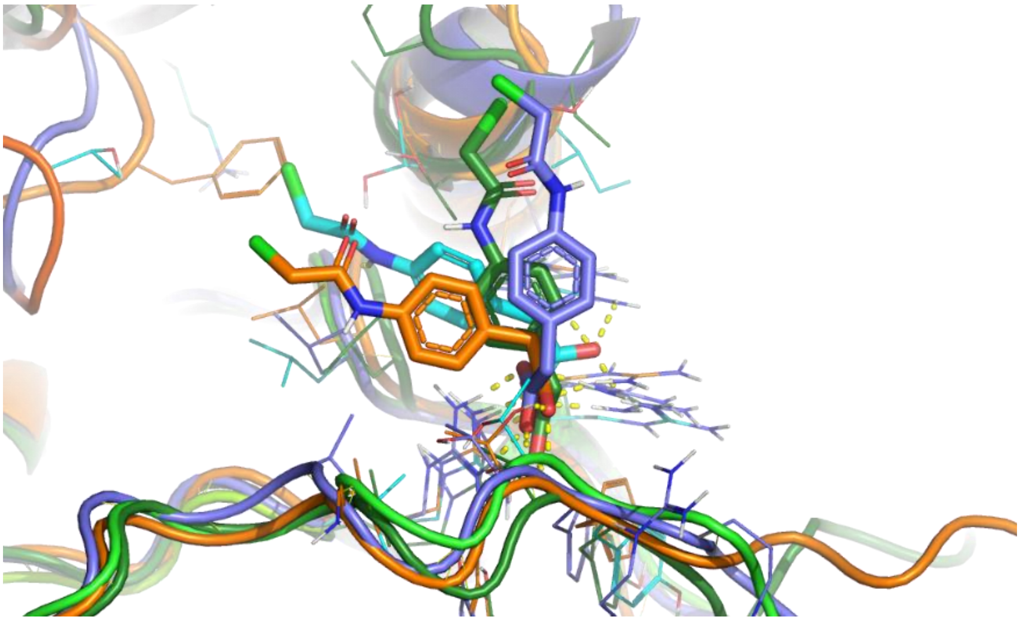

Figure S6. (A) The average RMSD plots for the 1903 within 1903-CDK9 complex during the three independent MD replicas (replica1:orange, replica2: blue and replica3: dark green). (B) MD snapshot structures at 200ns. Carbon atoms of replica1, replica2, replica2 and initial structure are shown in orange, purple, dark green and cyan, respectively. All nonpolar hydrogen atoms of receptor residues are omitted for clarify. Hydrogen bonding and salt bridge to side chain guanidino group of arginine residues are depicted by yellow dots.
